# Supplementary material for: The Fall Armyworm, Spodoptera frugiperda (Lepidoptera: Noctuidae), Influences Nilaparvata lugens Population Growth Directly, by Preying on Its Eggs, and Indirectly, by Inducing Defenses in Rice
Source: Int J Mol Sci. 2023 May 15;24(10):8754. doi: 10.3390/ijms24108754 (PMC10217797; doi:10.3390/ijms24108754)
Supplement: Supplementary file 1 [file ijms-24-08754-s001.zip › ijms-2366674-supplementary.pdf]

International Journal of *Molecular Sciences*

MDPI

Supplementary data

The Fall Armyworm, *Spodoptera frugiperda* (Lepidoptera: Noctuidae), Influences *Nilaparvata lugens* Population Growth Directly, by Preying on Its Eggs, and Indirectly, by Inducing Defenses in Rice

Chen Qiu [1], Jiamei Zeng [1], Yingying Tang [1], Qing Gao [1], Wenhan Xiao [1] and Yonggen Lou [1,2,*]

[1] State Key Laboratory of Rice Biology & Ministry of Agriculture Key Laboratory of Agricultural Entomology, Key Laboratory of Biology of Crop Pathogens and Insects of Zhejiang Province, Institute of Insect Sciences, Zhejiang University, Hangzhou 310058, China; cqiu2019@163.com (C.Q.); tsengchiamei@zju.edu.cn (J.Z.); tyy034899@zju.edu.cn (Y.T.); 11916008@zju.edu.cn (Q.G.); whxiao@zju.edu.cn (W.X.)
[2] Hainan Institute, Zhejiang University, Sanya 572025, China
* Correspondence: yglou@zju.edu.cn; Tel.: +86-571-8898-2622

Figure S1. Effects of rice plants that were infested by FAW larvae on the feeding, fecundity and developmental duration of BPH.

Figure S2. Pre-infestation of FAW larvae on rice plants does not influence the hatching rate and developmental duration of BPH eggs.

Figure S3. Pre-infestation of gravid BPH females on rice plants does not influence the growth of FAW larvae.

Figure S4. Effect of FAW larvae infestation on the attractiveness of BPH-infested plants to the parasitoid *Anagrus nilaparvatae*.

Figure S5. The experimental setup for BPH infestation (a), FAW infestation (b) and BPH honeydew excretion (c).

Table S1. Student's *t*-test analysis with data from figures in supporting information.

Table S2. Student's *t*-test analysis with data from Figure 1.

Table S3. Analysis of variance with data from Figure 1.

Table S4. Student's *t*-test analysis with data from Figure 2.

Table S5. Student's *t*-test analysis with data from Figure 3.

Table S6. Student's *t*-test analysis with data from Figure 4.

Table S7. Mean levels (+SE, n=5~6) of other phenolamides in rice plants that had been infested with 10 gravid BPH females for 24 h, after which the BPHs had been removed; levels measured 1, 3, 5 and 7 d after plants had been individually infested with either three 2nd-instar FAW larvae (BPH+FAW) or no FAW larvae (BPH).

Table S8. Mean levels (+SE, n=5~6) of other flavonoids in rice plants that had been infested with 10 gravid BPH females for 24 h, after which the BPHs had been removed; levels measured 1, 3, 5 and 7 d after plants had been individually infested with either three 2nd-instar FAW larvae (BPH+FAW) or no FAW larvae (BPH).

Table S9. Student's *t*-test analysis with data from Table S7.

Table S10. Student's *t*-test analysis with data from Table S8.

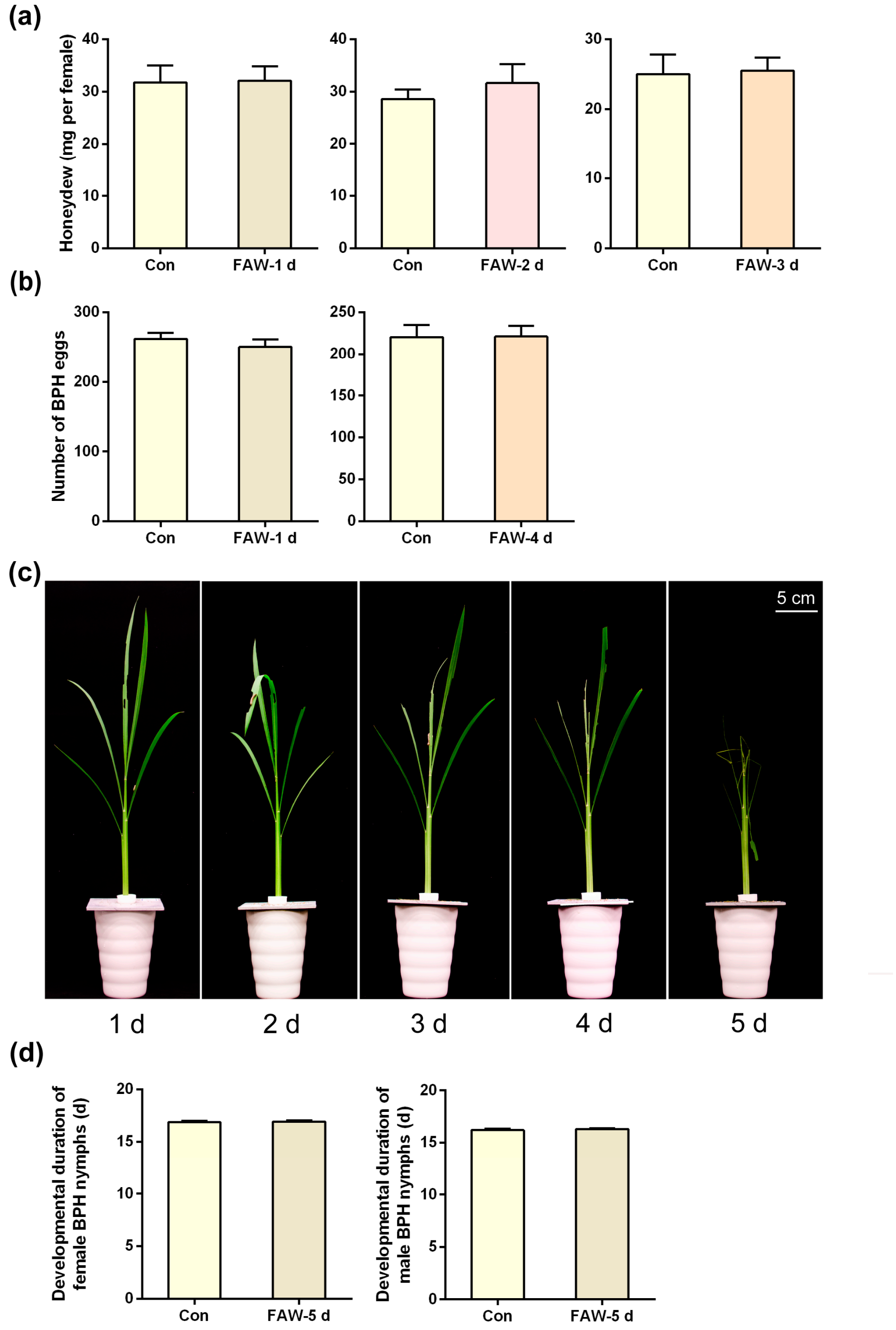

Figure S1. Effects of rice plants that were infested by FAW larvae on the feeding, fecundity and developmental duration of BPH.

(a) Mean mass of honeydew (+SE, n=21-25) excreted by a newly emerged BPH female adult feeding for 24 h on non-infested plants (Con) or plants that had been infested by one 3rd-instar FAW larva for 1, 2 or 3 d (FAW-1d, FAW-2d or FAW-3d); (b) Mean number of eggs (+SE, n=21-26) laid by a newly emerged female adult after feeding for 10 d on non-infested plants (Con) or plants that had been infested by one 3rd-instar FAW larva for 1 d or 4 d (FAW-1d or FAW-4d); (c) Phenotypes of rice plants that had been infested by one 3rd-instar FAW larva for 1-5 d; (d) Mean developmental duration (+SE, n=8~9) of BPH female or male nymphs on non-infested plants (Con) or plants that had been infested by one 3rd-instar FAW for 5 d (FAW-5d).

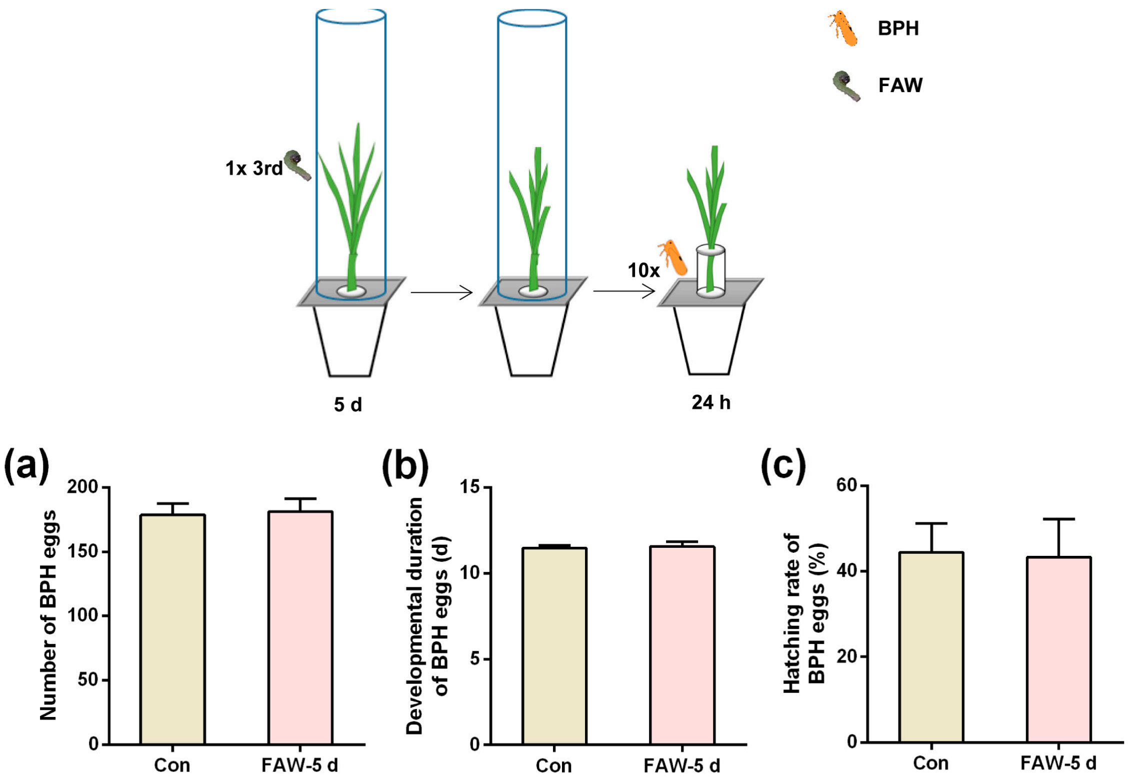

Figure S2. Pre-infestation of FAW larvae on rice plants does not influence the hatching rate and developmental duration of BPH eggs. Upper inset, schematic representation of the experimental design.

(a) Mean number of eggs (+SE, n=9~10) laid by 10 gravid BPH females for 24 h on non-infested plants (Con) or plants that had been pre-infested by one 3rd-instar FAW larva for 5 d (FAW-5d); (b) Mean developmental duration (+SE, n=10) of BPH eggs on non-infested plants (Con) or plants that had been pre-infested by one 3rd-instar FAW larva for 5 d (FAW-5d); (c) Mean hatching rate of BPH eggs (+SE, n=10) on non-infested plants (Con) or plants that had been pre-infested by one 3rd-instar FAW larva for 5 d (FAW-5d).

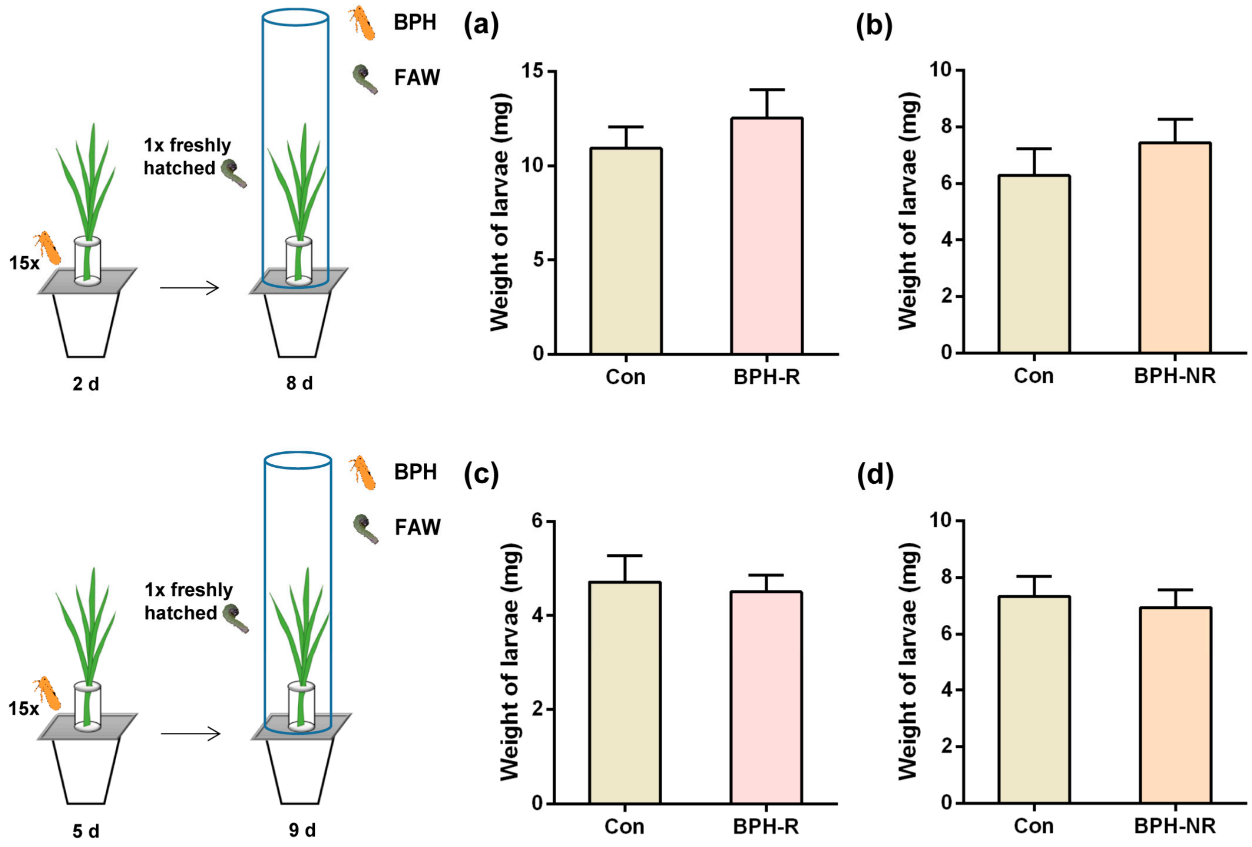

Figure S3. Pre-infestation of gravid BPH females on rice plants does not influence the growth of FAW larvae. Insets, schematic representations of the experimental design.

(a, b) Mean mass of individual FAW newly hatched larvae (+SE) 8 d after they fed on non-infested plants (Con) or plants that had been pre-infested by 15 gravid BPH females for 2 d, after which the BPHs had been removed (BPH-R) (a, n=12-14) or not removed (BPH-NR) (b, n=12-13); (c, d) Mean mass of individual FAW newly hatched larvae (+SE, n=21-28) 9 d after they fed on non-infested plants (Con) or plants that had been pre-infested by 15 gravid BPH females for 5 d, after which the BPHs had been removed (BPH-R) (c, n=21-28) or not removed (BPH-NR)(d, n=12-22).

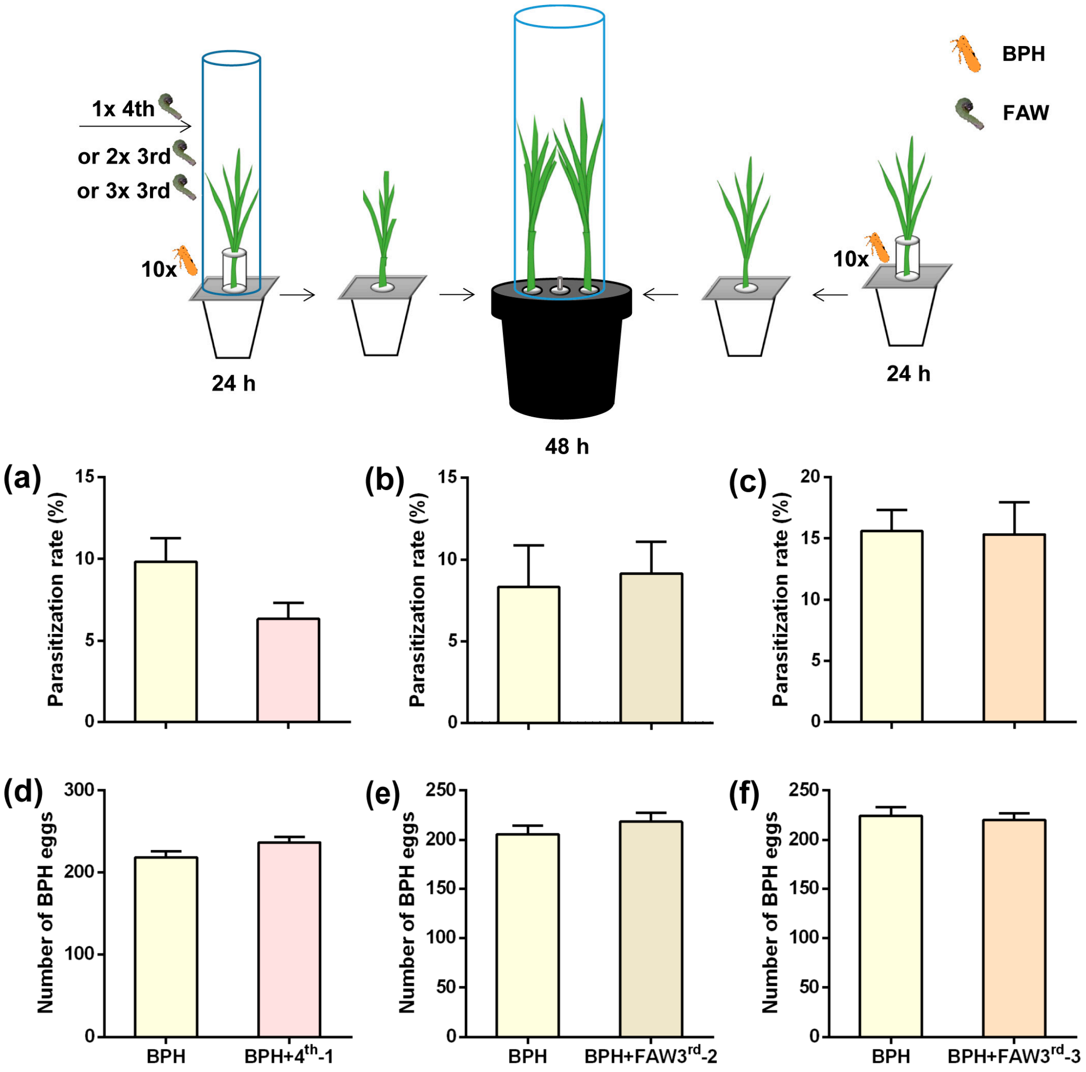

Figure S4. Effect of FAW larvae infestation on the attractiveness of BPH-infested plants to the parasitoid *Anagrus nilaparvatae*.

(a-c) Mean parasitism rate of BPH eggs (+SE ; a & b, n=15; c, n=19) by *A. nilaparvatae* on plants that had been infested with 10 gravid BPH females for 24 h and plants that had been co-infested with 10 gravid BPH females and one 4th-instar FAW larva (FAW4th-1) (a), two 3rd-instar FAW larvae (FAW3rd-2) (b) or three 3rd-instar FAW larvae (FAW3rd-3) (c) for 24 h. (d-f) Mean number of BPH eggs (+SE; d & e, n=15; f, n=19) laid by gravid BPH females on plants that had been infested with 10 gravid BPH females for 24 h and plants that had been co-infested with 10 gravid BPH females and one 4th-instar FAW larva (FAW4th-1) (d), two 3rd-instar FAW larvae (FAW3rd-2) (e) or three 3rd-instar FAW larvae (FAW3rd-3) (f) for 24 h. Upper inset, schematic representation of the experimental design.

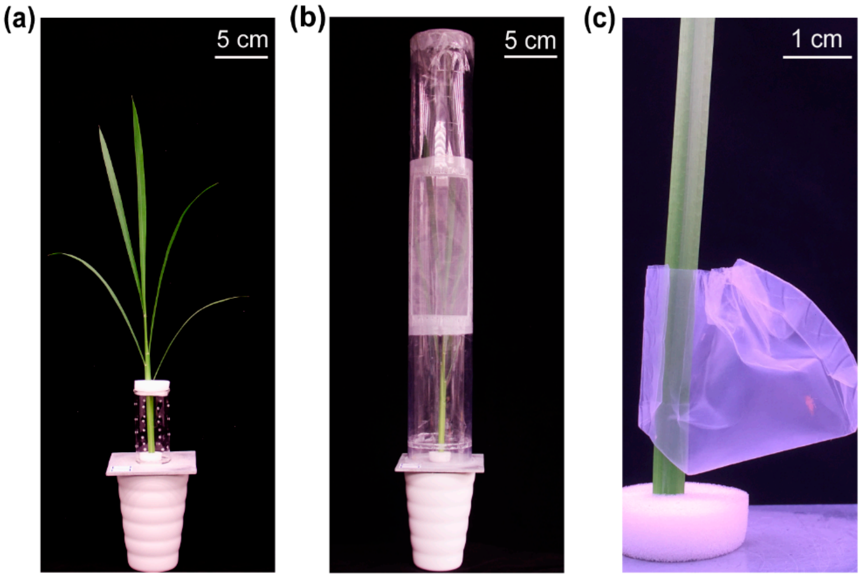

Figure S5. The experimental setup for BPH infestation (a), FAW infestation (b) and BPH honeydew excretion (c).

Table S1. Student's *t*-test analysis with data from figures in supporting information

Figure	Parameter		*t*	*df*	*P value*
Figure S1a	Honeydew	1 d	0.07461	48	0.9408
		2 d	0.0700	44	0.4876
		3 d	0.1413	46	0.8883
Figure S1b	Number of BPH eggs	1 d	0.8082	47	0.4230
		4 d	0.04185	40	0.9668
Figure S1c	Developmental duration of BPH nymphs	female	0.1469	15	0.8852
		male	0.6521	15	0.5242
Figure S2a	Number of BPH eggs		0.1676	17	0.8689
Figure S2b	Developmental duration of BPH eggs		0.2633	17	0.7954
Figure S2c	Hatching rate of BPH eggs		0.1045	17	0.9180
Figure S3a	Weight of larvae		0.8468	24	0.4054
Figure S3b	Weight of larvae		0.8963	23	0.3794
Figure S3c	Weight of larvae		0.3705	32	0.7134
Figure S3d	Weight of larvae		0.3355	47	0.7387
Figure S4a	Parasitization rate		1.975	28	0.0582
Figure S4b	Parasitization rate		0.2616	28	0.7955
Figure S4c	Parasitization rate		0.08505	36	0.9327
Figure S4d	Number of BPH eggs		1.829	28	0.0781
Figure S4e	Number of BPH eggs		1.015	28	0.3188
Figure S4f	Number of BPH eggs		0.4030	36	0.6893

Table S2. Student's *t*-test analysis with data from Figure 1

Figure	Parameter	*t*	*df*	*P* value
Figure 1a	Developmental duration of BPH eggs	0.5253	17	0.6061
Figure 1b	Hatching rate of BPH eggs	1.180	17	0.2544
Figure 1c	Number of BPH eggs	1.025	17	0.3196

Table S3. Analysis of variance with data from Figure 1

Figure	Parameter	*F*	*df*	*P* value
Figure 1d	Developmental duration of BPH eggs	5.814	2, 26	0.0082
Figure 1e	Hatching rate of BPH eggs	0.8744	2, 26	0.4290
Figure 1f	Number of BPH eggs	0.07442	2, 26	0.9285

Table S4. Student's *t*-test analysis with data from Figure 2

Figure	Parameter		*t*	*df*	*P* value
Figure 2a	Weight of larvae		3.8756	51	0.0003
Figure 2b	Inceased percentage of mass of individual larvae	1 d	2.372	42	0.0224
		3 d	12.02	39	<0.0001
Figure 2c	Number of BPH eggs		6.330	58	<0.0001
Figure 2d	Inceased percentage of mass of individual larvae	2 d	2.523	56	0.0145
		3 d	2.991	56	0.0041

Table S5. Student's *t*-test analysis with data from Figure 3

Compounds		*t*	*df*	*P* value
JA	Con	0	8	>0.9999
	1 d	1.061	7	0.3241
	3 d	1.564	8	0.1563
	5 d	0.5392	8	0.6044
	7 d	1.074	8	0.3143
JA-ILe	Con	0	8	>0.9999
	1 d	0.5772	7	0.5819
	3 d	3.085	8	0.0150
	5 d	0.2407	8	0.8158
	7 d	1.114	8	0.2976
SA	Con	0	8	>0.9999
	1 d	1.707	7	0.1315
	3 d	1.045	8	0.3264
	5 d	0.6932	8	0.5078
	7 d	1.766	8	0.1155
ABA	Con	0	8	>0.9999
	1 d	0.5090	7	0.6264
	3 d	2.982	8	0.0175
	5 d	1.483	8	0.1765
	7 d	0.9744	8	0.3584
H_2O_2	Con	0	8	>0.9999
	1 d	0.2947	8	0.7757
	3 d	1.322	8	0.2227
	5 d	0.5505	8	0.5970
	7 d	0.1938	8	0.8512

Table S6. Student's *t*-test analysis with data from Figure 4

Compounds		*t*	*df*	*P* value
N-feruloylputrescine	1 d	2.591	9	0.0291
	3 d	0.3461	9	0.7372
	5 d	1.186	10	0.2630
	7 d	1.001	10	0.3405
luteolin	1 d	2.313	9	0.0460
	3 d	1.897	9	0.0904
	5 d	0.3899	10	0.7048
	7 d	5.308	10	0.0003
asrtagalin	1 d	2.594	9	0.0290
	3 d	1.108	9	0.2968
	5 d	0.6745	10	0.5153
	7 d	3.905	10	0.0029
luteolin 7-O-glucoside	1 d	2.542	9	0.0316
	3 d	1.010	9	0.3388
	5 d	0.9921	10	0.3445
	7 d	3.057	10	0.0121
schaftoside+isoschaftoside	1 d	5.301	9	0.0005
	3 d	0.5253	9	0.6120
	5 d	1.410	10	0.1888
	7 d	1.178	10	0.2662

Table S7. Mean levels (+SE, n=5~6) of other phenolamides in rice plants that had been infested with 10 gravid BPH females for 24 h, after which the BPHs had been removed; levels measured 1, 3, 5 and 7 d after plants had been individually infested with either three 2nd-instar FAW larvae (BPH+FAW) or no FAW larvae (BPH)

		1d		3d		5d		7d	
No.	Compounds (ng/g)	BPH	BPH+FAW	BPH	BPH+FAW	BPH	BPH+FAW	BPH	BPH+FAW
1	Cinnamyl putrescine	2.406 ± 0.9023	1.622 ± 0.2873	0.9260 ± 0.07708	1.271 ± 0.1926	0.8753 ± 0.1167	0.8257 ± 0.07191	0.6377 ± 0.09556	0.8122 ± 0.08717
2	P-coumaryl putrescine	715.6 ± 104.6	526.1 ± 18.55	316.7 ± 28.30	352.5 ± 64.07	234.7 ± 29.56	200.1 ± 14.90	104.5 ± 22.80	266.5 ± 90.19
3	P-coumarylguanbutylamine	35.46 ± 1.952	44.26 ± 6.132	28.92 ± 2.740	32.01 ± 5.260	26.65 ± 3.247	20.08 ± 0.8487	20.08 ± 0.8586	34.68 ± 8.088
4	Myrosinyl putrescine	27.29 ± 7.684	25.39 ± 4.266	47.07 ± 9.982	47.61 ± 4.332	45.21 ± 5.863	63.23 ± 16.86	98.07 ± 20.36	72.58 ± 22.51
5	Feruloylagmatine	7.171 ± 0.7346	7.335 ± 0.8474	6.407 ± 0.4706	6.888 ± 0.5885	7.095 ± 1.052	4.994 ± 0.2212	5.460 ± 0.5562	6.923 ± 0.9817
6	Feruloyltyramine	8.287 ± 2.844	4.096 ± 0.8196	0.4436 ± 0.08006	0.5355 ± 0.03418	0.4029 ± 0.06166	0.4038 ± 0.1244	0.1585 ± 0.03638	1.123 ± 0.7530
7	Dicoumarimide	0.03126 ± 0.001673	0.03137 ± 0.001139	0.03248 ± 0.003125	0.03362 ± 0.002284	0.02971 ± 0.001168	0.03026 ± 0.0009077	0.02853 ± 0.0005904	0.02803 ± 0.0001852
8	Diferulimide	114.0 ± 40.80	127.0 ± 36.08	230.4 ± 50.42	302.7 ± 49.89	146.1 ± 38.88	389.8 ± 219.7	185.6 ± 53.04	285.6 ± 43.27

Table S8. Mean levels (+SE, n=5~6) of other flavonoids in rice plants that had been infested with 10 gravid BPH females for 24 h, after which the BPHs had been removed; levels measured 1, 3, 5 and 7 d after plants had been individually infested with either three 2nd-instar FAW larvae (BPH+FAW) or no FAW larvae (BPH).

		1d		3d		5d		7d	
No.	Compounds (ng/g)	BPH	BPH+FAW	BPH	BPH+FAW	BPH	BPH+FAW	BPH	BPH+FAW
1	Cosmosiin	15.71 ± 2.254	15.05 ± 1.585	10.46 ± 1.194	9.915 ± 0.8753	8.926 ± 0.4674	8.041 ± 0.6055	7.116 ± 0.3972	6.198 ± 0.6545
2	Isovitexin	5.710 ± 0.6574	6.567 ± 0.6014	5.392 ± 0.4986	5.041 ± 0.3118	4.294 ± 0.3003	4.320 ± 0.2999	3.334 ± 0.1617	3.283 ± 0.1782
3	Prunin	7.458 ± 0.9341	5.225 ± 0.5657	4.458 ± 0.2446	4.143 ± 0.5099	3.459 ± 0.1914	2.992 ± 0.3674	2.387 ± 0.2308	2.312 ± 0.2199
4	Isoquercitrin	1.820 ± 0.3011	2.150 ± 0.2230	1.943 ± 0.1811	1.700 ± 0.1575	1.679 ± 0.08957	1.583 ± 0.1616	1.322 ± 0.06077	1.153 ± 0.08415
5	Neoschaftoside	0.2504 ± 0.03500	0.2828 ± 0.04805	0.1408 ± 0.01852	0.1172 ± 0.01470	0.1266 ± 0.01462	0.1165 ± 0.007548	0.08307 ± 0.005845	0.07611 ± 0.01867
6	Carlinoside	4.848 ± 0.6683	4.286 ± 0.6634	2.465 ± 0.1562	1.628 ± 0.3891	1.519 ± 0.3141	1.823 ± 0.1101	3.149 ± 0.8618	3.211 ± 1.211
7	Rutin	16.35 ± 2.410	17.88 ± 1.178	14.90 ± 0.9853	13.27 ± 0.9147	12.02 ± 0.6208	11.38 ± 1.459	10.09 ± 0.6204	10.18 ± 1.345

Table S9. Student's *t*-test analysis with data from Table S7

Compounds		*t*	*df*	*P* value
Cinnamyl putrescine	1 d	0.7726	9	0.4596
	3 d	1.539	9	0.1581
	5 d	0.3691	10	0.7250
	7 d	1.349	10	0.2070
P-coumaryl putrescine	1 d	1.962	9	0.0814
	3 d	0.4754	9	0.6458
	5 d	1.047	10	0.3200
	7 d	2.2037	10	0.0521
P-coumarylguanbutylamine	1 d	1.0700	9	0.3125
	3 d	0.4887	9	0.6367
	5 d	1.959	10	0.0785
	7 d	1.794	10	0.1030
Myrosinyl putrescine	1 d	0.2265	9	0.8259
	3 d	0.05301	9	0.9589
	5 d	0.8586	10	0.4107
	7 d	0.8401	10	0.4205
Feruloylagmatine	1 d	0.1432	9	0.8893
	3 d	0.6193	9	0.5511
	5 d	1.953	10	0.0794
	7 d	1.296	10	0.2239
Feruloyltyramine	1 d	1.539	9	0.1581
	3 d	1.127	9	0.2890
	5 d	0.006389	10	0.9950
	7 d	2.1202	10	0.0600
Dicoumarimide	1 d	0.06016	9	0.9533
	3 d	0.3024	9	0.7692
	5 d	0.3683	10	0.7203
	7 d	0.8197	10	0.4315
Diferulimide	1 d	0.2396	9	0.8160
	3 d	1.011	9	0.3383
	5 d	1.0387	10	0.3234
	7 d	1.460	10	0.1749

Table S10. Student's *t*-test analysis with data from Table S8

Compounds		*t*	*df*	*P* value
Cosmosiin	1 d	0.2471	9	0.8103
	3 d	0.3767	9	0.7151
	5 d	1.157	10	0.2740
	7 d	1.199	10	0.2582
Isovitexin	1 d	0.9266	9	0.3611
	3 d	0.6184	9	0.5516
	5 d	0.06132	10	0.9523
	7 d	0.2119	10	0.8364
Prunin	1 d	2.127	9	0.0623
	3 d	0.5207	9	0.6152
	5 d	1.127	10	0.2861
	7 d	0.2355	10	0.8186
Isoquercitrin	1 d	0.8988	9	0.3922
	3 d	1.017	9	0.3359
	5 d	0.5225	10	0.6127
	7 d	1.622	10	0.1358
Neoschaftoside	1 d	0.5248	9	0.6124
	3 d	1.013	9	0.3373
	5 d	0.6186	10	0.5500
	7 d	0.3556	10	0.7295
Carlinoside	1 d	0.5922	9	0.5683
	3 d	1.848	9	0.0977
	5 d	0.8439	9	0.4206
	7 d	0.05197	10	0.9596
Rutin	1 d	0.6028	9	0.5615
	3 d	1.210	9	0.2571
	5 d	0.4059	10	0.6974
	7 d	0.05743	10	0.9553
